# A Web-Based Intervention to Prevent Multiple Chronic Disease Risk Factors Among Adolescents: Co-Design and User Testing of the Health4Life School-Based Program

**DOI:** 10.2196/19485

**Published:** 2020-07-28

**Authors:** Katrina Elizabeth Champion, Lauren Anne Gardner, Cyanna McGowan, Cath Chapman, Louise Thornton, Belinda Parmenter, Nyanda McBride, David R Lubans, Karrah McCann, Bonnie Spring, Maree Teesson, The Health4Life Team, Nicola Clare Newton

**Affiliations:** 1 The Matilda Centre for Research in Mental Health and Substance Use The University of Sydney Sydney Australia; 2 Department of Preventive Medicine Northwestern University Feinberg School of Medicine Chicago, IL United States; 3 Department of Exercise Physiology, School of Medical Sciences, Faculty of Medicine UNSW Sydney Sydney Australia; 4 National Drug Research Institute, Faculty of Health Sciences Curtin University Perth Australia; 5 Priority Research Centre for Physical Activity and Nutrition, Faculty of Education and Arts University of Newcastle Callaghan Australia; 6 See Authors' Contributions section

**Keywords:** primary prevention, schools, eHealth, chronic disease, mobile phone, health promotion

## Abstract

**Background:**

Chronic diseases are the leading cause of death worldwide. Addressing key lifestyle risk factors during adolescence is critical for improving physical and mental health outcomes and reducing chronic disease risk. Schools are ideal intervention settings, and electronic health (eHealth) interventions afford several advantages, including increased student engagement, scalability, and sustainability. Although lifestyle risk behaviors tend to co-occur, few school-based eHealth interventions have targeted multiple behaviors concurrently.

**Objective:**

This study aims to summarize the co-design and user testing of the Health4Life school-based program, a web-based cartoon intervention developed to concurrently prevent 6 key lifestyle risk factors for chronic disease among secondary school students: alcohol use, smoking, poor diet, physical inactivity, sedentary recreational screen time, and poor sleep (the *Big 6*).

**Methods:**

The development of the Health4Life program was conducted over 18 months in collaboration with students, teachers, and researchers with expertise relevant to the Big 6. The iterative process involved (1) scoping of evidence and systematic literature review; (2) consultation with adolescents (N=815) via a cross-sectional web-based survey to identify knowledge gaps, attitudes, barriers, and facilitators in relation to the Big 6; (3) content and web development; and (4) user testing of the web-based program with students (n=41) and teachers (n=8) to evaluate its acceptability, relevance, and appeal to the target audience.

**Results:**

The co-design process resulted in a six-module, evidence-informed program that uses interactive cartoon storylines and web-based delivery to engage students. Student and teacher feedback collected during user testing was positive in terms of acceptability and relevance. Commonly identified areas for improvement concerned the length of modules, age appropriateness of language and alcohol storyline, the need for character backstories and links to syllabus information, and feasibility of implementation. Modifications were made to address these issues.

**Conclusions:**

The Health4Life school-based program is the first universal, web-based program to concurrently address 6 important chronic disease risk factors among secondary school students. By adopting a multiple health behavior change approach, it has the potential to efficiently modify the Big 6 risk factors within one program and to equip young people with the skills and knowledge needed to achieve and maintain good physical and mental health throughout adolescence and into adulthood.

## Introduction

### Background

Chronic diseases such as cardiovascular disease, type 2 diabetes, and mental disorders are the leading causes of death globally [1]. It is well established that major chronic diseases share 4 common lifestyle risk factors: poor diet, physical inactivity, smoking, and alcohol use [1,2]. Emerging evidence has also linked these established chronic disease risk factors with 2 additional risk behaviors: sedentary behavior (ie, sitting and recreational screen time) [3,4] and poor sleep (ie, long or short duration and/or poor quality) [5]. Together, these 6 risk factors (the *Big 6*) are important targets for prevention and early intervention programs to reduce chronic disease.

A life course approach to prevention includes intervening early to reduce risk factors *before the onset* of chronic disease [6]. Early adolescence is a critical period to intervene, as it coincides with the emergence of many health risk factors and is a time when young people acquire greater autonomy over their lifestyle choices [7]. To date, most prevention approaches have focused on changing single behaviors, despite risk factors commonly co-occurring [8]. For example, watching television is associated with high-fat snacking [9], and young people who engage in risky alcohol and other drug use are also more likely to eat poorly and be sedentary [7]. In recognition of this clustering, multiple health behavior change interventions [10] have been developed to address risk factors together, rather than in isolation. These interventions are underpinned by the *transfer theory* [11], whereby skills and knowledge learned about one behavior are thought to transfer to other contexts [12], resulting in improvements across multiple behaviors without additional intervention [13]. For example, an intervention targeting physical activity was also shown to improve eating habits [14].

Schools are ideal locations to deliver healthy lifestyle interventions, as they provide an opportunity to engage large numbers of students during this critical time period, and in Australia, schools are mandated to teach health education. Furthermore, outside of the family environment, the school is the primary setting within which the development of children and young people can be directed and shaped [15]. As teaching time is often limited, multiple health behavior change interventions that can simultaneously address multiple risk factors are particularly advantageous. However, evidence-based prevention programs are typically poorly implemented, often because of limited resources, a lack of teacher training and support, and program adaptations that undermine efficacy [16,17]. In fact, it has been estimated that only 14% of programs delivered in schools have the correct content and modes of delivery [18], and more recently, that teachers adapt between 50% and 68% of content in prevention interventions [17,19]. This may help to explain why interventions targeting health behaviors, such as physical activity, among adolescents have been largely unsuccessful [20]. Web-based interventions have the potential to overcome implementation barriers encountered by face-to-face interventions and offer several advantages, including increased student engagement, scalability, and program fidelity [21]. For example, core content is preprogrammed on the web, and completion is self-directed by students; therefore, delivery is not dependent on teacher training or skills, which promotes faithful delivery of key program components [22,23]. Research indicates that web-based interventions can be an effective means of preventing and reducing substance use among adolescents [21,24], and computer-based education has been shown to be superior to a generic classroom curriculum in increasing physical activity and improving diet [25]. Furthermore, schools are increasingly looking for digital web-based platforms to deliver key curriculum content as an alternative or adjunct to face-to-face teaching. However, there are no existing web-based interventions that adopt a multiple health behavior change approach to address the Big 6 among school students.

The effective *Climate Schools* prevention programs [26-28] use interactive, web-based cartoons about a group of teenagers to engage students; summaries and optional class activities to reinforce key content; and principles of social influence theory, namely, information provision, normative education, and resistance skills training [29], to prevent risky substance use and associated harms among adolescents [26-28]. Using the *Climate Schools* programs as a model, we developed the Health4Life school-based program to address gaps in the field. Underpinned by a multiple health behavior change approach [10], Health4Life aims to empower adolescents to reduce chronic disease risk and improve current physical and mental health by providing simultaneous web-based education about the Big 6 lifestyle risk factors. The intervention consists of 3 components: (1) a school-based program delivered to all grade 7 students (aged approximately 12 years) during class time, regardless of risk (universal prevention); (2) an accompanying smartphone app, available to all students regardless of risk (universal prevention); and (3) additional web and app content delivered outside of school to students who remain at risk of chronic disease 1 and 2 years after initial intervention delivery (selective prevention).

### Objectives

This study aims to describe the co-design and user testing of the universal in-class school-based component of the Health4Life intervention to aid in the development of future web-based prevention resources.

## Methods

### Co-Design

**Figure 1:**
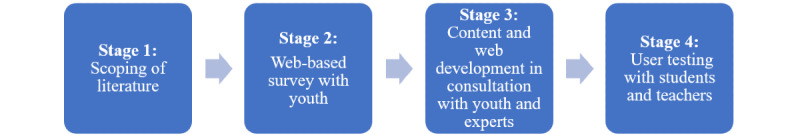
Co-design of the Health4Life school-based program.

The Health4Life school-based program was developed using an iterative co-design process consisting of 4 key stages (Figure 1). A co-design approach was used to engage young people in the co-creation and refinement of the program. Co-design allows for a richer understanding of user needs and may be particularly important in fostering the engagement and satisfaction needed for web-based interventions to succeed among young people [30]. The Health4Life school-based program was co-designed with youth and teachers, with development coordinated by a research team comprising experts in addiction, physical activity, exercise physiology, sleep, dietetics, mental health, electronic health (eHealth) interventions, and behavior change.

### Stage 1: Scoping of the Literature

Although guided by an overarching multiple health behavior change approach [10], scoping of the literature was conducted to identify evidence-based prevention principles and best practice for the prevention of each of the Big 6 behaviors. Specifically, members of the research team with expertise in each of the 6 health behaviors were consulted to identify key behavioral theories and seminal school-based prevention papers and systematic reviews concerning each behavior. In addition, searches were conducted to identify national guidelines or recommendations, along with any further school-based prevention papers or systematic reviews and behavioral theories that could guide development. The key behavioral theories and evidence-based prevention principles that were identified and embedded in the Health4Life school-based program are outlined in Table 1.

**Table 1:** Key behavioral theories and evidence-based prevention principles for each of the Big 6 behaviors.

Big 6 behavior	Key behavioral theories	Evidence-based prevention principles
Alcohol use and smoking	Social influence theory Social learning theory Social cognitive theory	Normative education Resistance skills training Information and knowledge provision Using peer leaders Harm minimization Life skills training, for example, decision making, problem-solving, coping skills, refusal and assertion skills, self-esteem, and self-control
Physical activity	Self-determination theory Social cognitive theory	Development of competence, relatedness and social connection, and autonomy Promotion of autonomous motivation (engaging in behavior that is valued, personally relevant, and enjoyable) Self-regulatory skill development, for example, goal setting, self-monitoring, and decision making
Sedentary recreational screen time	Self-determination theory Social cognitive theory	Development of competence, relatedness and social connection, and autonomy Self-regulatory skill development, for example, goal setting, self-monitoring, and decision making
Sleep	Two-process model of sleep Social cognitive theory	Teaching biological contributing factors to sleep timing and duration Maintaining regular sleep patterns and identifying sleep problems Self-regulatory skill development, for example, goal setting, self-monitoring, and decision making
Diet	Social cognitive theory	Self-regulatory skill development, for example, goal setting, self-monitoring, and decision making

In addition, to better understand the effectiveness of interventions targeting multiple risk behaviors, we conducted a systematic review and meta-analysis of eHealth school-based multiple health behavior change interventions targeting two or more of the Big 6 risk factors [31]. A total of 22 publications assessing 16 interventions were included in the review. Most of the studies assessed interventions to prevent alcohol use and smoking or improve diet and physical activity. Few studies targeted screen or sitting time, and none addressed sleep. Pooled findings supported the effectiveness of eHealth school-based multiple health behavior change interventions for improving both accelerometer- (standard mean difference [SMD] 0.33; 95% CI 0.05 to 0.61) and self-report–(SMD 0.14; 95% CI 0.05 to 0.23) measured physical activity, screen time (SMD −0.09; 95% CI −0.17 to −0.01), and fruit and vegetable intake (SMD 0.11; 95% CI 0.03 to 0.19). However, the effects were small and short lived. No effects were seen for alcohol or smoking, fat, or sugar-sweetened beverage or snack consumption.

Interventions varied greatly in their content and delivery. Few studies described specific behavior change techniques, and none of the studies referred to an established behavior change taxonomy [32], making it difficult to tease apart effective intervention components that could be embedded into future programs. However, effective programs tended to use computer-based tailored feedback, whereby students were provided with individualized, normative, or stage-matched feedback based on self-reported responses to computer-based assessments, suggesting that this may be an important component to include in future interventions.

Results from the systematic review highlighted the need to develop multiple health behavior change interventions that address sleep problems among youth, as none of the included interventions addressed sleep. This is despite the growing recognition that physical inactivity, sedentary behavior, and sleep are codependent [33,34] and the fact that many young people report sleep problems [35]. Generally, ineffective interventions for alcohol use and smoking were brief, primarily used the transtheoretical model [36], and did not provide sufficient opportunities for skill building. It was concluded that future eHealth multiple health behavior change interventions targeting alcohol and tobacco use alongside other risk behaviors should be guided by principles of effective substance use prevention (eg, normative education and life skills training). Finally, the findings indicated that eHealth multiple behavior change interventions were only effective in increasing physical activity among students aged 13 years or older, and boys had a greater increase in physical activity than girls. This suggests that such interventions might not adequately engage girls or young teenagers (aged <13 years) and that formative research is required to better understand the beliefs, attitudes, and motivations of these groups regarding physical activity.

### Stage 2: Web-Based Survey With Adolescents

A web-based self-report survey was conducted to understand young people’s knowledge about the Big 6, their current engagement with health behaviors, their beliefs and attitudes about health, and barriers and facilitators to achieving good health.

#### Participants and Procedure

A total of 7 independent secondary schools in New South Wales and the Australian Capital Territory of Australia were invited to participate. Of the 7 schools, 3 agreed to participate. Participating schools were asked to distribute information and consent forms to the parents or guardians of their grade 7 to 9 students. Passive parental consent and active student consent were required for youth to be eligible to participate in the study (99% consent rate). Students completed an anonymous web-based survey in a supervised classroom setting between August 2018 and September 2018. Participants who completed the survey were entered into the draw to win a Fitbit valued at Aus $450 (US $315), with one prize given per school. Ethics approval was obtained from the University of New South Wales Human Research Ethics Committee (HREC; HC180224).

#### Measures

Demographic data collected included age, sex, self-reported height and weight, and postcode. Self-reported health status was measured using a single item, *“*How would you rate your own health?,” with responses made on a 5-point Likert scale ranging from “Poor” to “Excellent.”

##### Physical Activity

To assess moderate-to-vigorous physical activity (MVPA) students were asked, *“*Over the past 7 days, how many days did you do moderate or vigorous intensity physical activity for at least 60 minutes per day?*”* [37]. Participants were provided with a written description of what constitutes MVPA.

##### Screen Time

Using a modified version of the Adolescent Sedentary Activity Questionnaire [38], students were asked to recall the amount of time (hours and minutes) typically spent on recreational screen time on weekdays and weekend days.

##### Fruit and Vegetable Consumption

Using validated short items commonly used in health research [39,40], fruit intake was assessed via a single item: “About how many serves of fruit do you usually have each day?*”* (“don’t eat fruit” to “6 serves or more”). A similar item measured vegetable intake. Participants were provided with information about what constitutes one serve of fruit or vegetables.

##### Sleep

Students were asked to report their usual bedtime and wake time on school nights and weekend nights. Sleep duration (in hours) was calculated as the difference between wake time and bedtime. Self-reported estimates of bedtime, wake time, and sleep duration have been shown to be reliable and valid [41].

##### Alcohol and Tobacco Use

Students were asked to report if they had ever tried alcohol or tobacco, based on validated items used in previous school-based trials [28,42].

##### Attitudes, Knowledge, Barriers, and Facilitators

A series of open-ended questions were asked to understand attitudes toward health, for example, *“*What does good health mean to you?” Students were asked 5 multiple-choice questions to test their knowledge about age-appropriate national recommendations for the Big 6 risk behaviors. These items were based on national guidelines for each behavior [34,43-45]. As there are no official national guidelines for smoking, participants’ knowledge could not be assessed. Open-ended questions were used to assess key motivational factors, barriers, and facilitators of health, for example, “What gets in the way, or stops you, from being more physically active?*”*

### Analysis

Data were collated and analyzed using IBM SPSS Statistics 24 (IBM Corp). Descriptive analyses were conducted to illustrate the sample characteristics, prevalence rates of the Big 6, and knowledge. For the collected open-ended data, we selected a subsample of student responses (20%-25%) and carried out a qualitative analysis on these responses until no new themes emerged (ie, data saturation was reached). The sample was stratified by age and year group, and a random subsample was selected to ensure balanced representation across age and year groups. Using an inductive approach, one author (LG) coded responses from the subsample, examined the data for frequent or significant responses, and grouped them according to key themes or categories.

#### Findings

##### Health Behaviors and Knowledge

A total of 815 students (mean age 13.89 years, SD 0.89; 84.3% [687/815] female) from 3 schools (2 coeducational and 1 female only) completed the survey. The majority of participants perceived their health to be “very good” (334/801, 41.7%) or “good” (274/801, 34.2%); however, adherence to national guidelines for the Big 6 was mixed. Only 12.9% (101/784) of participants achieved the recommended amount of MVPA, and only 11.8% (92/779) of participants reported eating enough vegetables per day. The majority of students (622/779, 79.8%) reported eating sufficient fruit serves, and approximately half of the participants (382/779, 49.0%) met guidelines for screen time on weekdays; however, only 23.0% (179/779) of participants met guidelines on the weekend. A small proportion of students (29/778, 3.7%) had tried tobacco in their lifetime, and 64.0% (498/779) of students had used alcohol in their lifetime (including a taste or sip). Among students aged 14 years or older, 70.1% (262/374) met the guidelines of sleeping 8 to 10 hours; however, only 53.0% (222/419) of students aged 12 to 13 years met their guideline of 9 to 11 hours. Knowledge of the recommended guidelines was poor for physical activity, diet, and sleep (Table 2); however, most students (719/786, 91.5%) correctly identified that for adolescents, the safest option is not to drink alcohol at all, and 98.1% (771/786) of students agreed that smoking is harmful.

**Table 2:** Percentage of students who correctly identified national guidelines.

Risk behavior and Australian guidelines for young people		Value, n (%)	
**Physical activity (n=798)**			
	Accumulate 60 min or more of moderate-to-vigorous physical activity per day		212 (26.6)
**Sedentary recreational screen time (n=792)**			
	No more than 2 hours per day		394 (49.7)
**Sleep**			
	9-11 hours of uninterrupted sleep per night for those aged 5-13 years (n=405)		77 (19.0)
	8-10 hours per night for those aged 14-17 years (n=351)		210 (59.8)
**Diet (n=789)**			
	Minimum of 2 serves of fruit per day		279 (35.4)
	Minimum of 5 serves of vegetables per day		266 (33.7)
**Alcohol use (n=786)**			
	For children and young people aged younger than 18 years, not drinking alcohol is the safest option		719 (91.5)

##### Attitudes, Barriers, and Facilitators

The key themes extracted from the open-ended responses are illustrated in Table 3. Many of the student responses aligned with the key behavioral theories and prevention principles outlined in stage 1. For example, consistent with self-determination theory, key facilitators of physical activity included social connection and intrinsic motivation. In addition, in line with social influence and social learning theories, the role of peers and older influences was prominent when considering reasons to drink alcohol and smoke cigarettes. These findings further shaped the storylines and guided the core content in the Health4Life program (more information is provided in the *Stage 3: Content and Web Development* section).

**Table 3:** Summary of key themes extracted from the open-ended responses of students.

Theme		Example	
**The meaning of good health**			
	Physical health factors such as being physically fit or active and having a good diet		“Eating well and just keeping active.” (Male, 14 years)
	Mental health factors such as having a positive mental state and feeling well		“Being active and eating healthy, but also to have a positive and healthy mindset.” (Female, 13 years)
	Emotional health factors such as feeling happy		“Good health to me means being happy. It also means being healthy by exercising and eating the right foods. But mainly I think it means being happy.” (Female, 13 years)
**Physical activity motivators**			
	Outcomes of being active such as the positive emotions felt afterward and maintenance of fitness and health		“I know that when I do exercise it puts me in a better mood which motivates me because who doesn't want to be in a good mood.” (Female, 14 years)
	Social factors such as friends, family, and teammates		“My friends and family. They help encourage me to take part in sport and also get me outside of the house for a run.” (Female, 12 years)
	Enjoyment and fun derived from physical activity and sports		“What motivates me is my liking and love for sports. I find most sports extremely fun to play.” (Female, 12 years)
**Physical activity barriers**			
	Lack of time because of school, homework, or assignments and other commitments		“My schedule is full with other work [homework, study for exams] and also I have commitments like family and community work so I don’t always have enough time to work out.” (Male, 14 years)
	Laziness or a lack of motivation and feeling tired		“Things such as not having enough sleep, not being motivated enough and being too tired can contribute.” (Female, 13 years)
**Reasons for young people smoking**			
	To look or feel cool		“It is seen as the cool thing to do or a way to make you seem older than you are.” (Male, 14 years)
	Peer pressure or to fit in		“Peer pressure from people either older than them or their age.” (Female, 12 years)
	Curiosity or just to try it		“Some people my age smoke probably because they want to seem like a ‘cool’ kid and also try to experience what it tastes like.” (Female, 14 years)
	No reason as people do not smoke at that age		“I don’t think they smoke.” (Female, 12 years)
**Reasons for young people drinking alcohol**			
	To look or feel cool		“I think people my age drink alcohol because they want to seem cool and mature, as alcohol is an adult thing to do.” (Female, 13 years)
	Peer pressure or to fit in		“Because of peer pressure, thinks it’s cool and will achieve friends loyalty.” (Male, 13 years)
	Curiosity		“People my age drink alcohol mostly out of curiosity and because they want to see what it tastes like.” (Female, 12 years)
	No reason as people do not drink alcohol at that age		“I don't think people my age drink alcohol.” (Female, 13 years)
**Barriers to getting enough sleep**			
	Using technology and devices		“...because they are on their technology just before bed, making it hard to get to sleep.” (Female, 13 years)
	Other commitments taking their time such as homework or extracurricular activities		“I think that people my age do not sleep because they stay awake doing homework or schoolwork. Assignments, projects.” (Male, 14 years)

### Stage 3: Content and Web Development

#### Background and Content Development

The development of the Health4Life school-based program was based on the effective *Climate Schools* substance use prevention programs [26-28], which use web-based cartoon storylines about a group of teenagers to deliver key prevention messages and engage students. Underpinned by a multiple health behavior change approach [10] and guided by the key behavioral theories and prevention principles for each of the Big 6 behaviors identified in stage 1, the findings from the web-based youth survey conducted in stage 2, and consultation with researchers with expertise in the Big 6, the core content areas were developed, which formed the basis for the 6-module Health4Life program (Table 4).

**Table 4:** Summary of Health4Life program content.

Module	Key messages
1	Guidelines for eating healthily and benefits of a healthy diet Tips for increasing water intake Sleep needs of adolescents and the benefits of sleeping well Guidelines for recreational screen time and the benefits of limiting screen use
2	Prevalence and patterns of alcohol and tobacco use among adolescents Australian guidelines to reduce health risks from drinking alcohol Identifying reasons why teenagers choose to, or not to, drink alcohol Benefits of being physically active Finding physical activities that you enjoy
3	Short- and long-term consequences of alcohol and tobacco use Consequences of excessive sedentary recreational screen time Strategies for reducing sedentary recreational screen time Responsible use of social media
4	Social, financial, and legal consequences of alcohol and tobacco use Assertive communication skills and refusal skills Australian guidelines for physical activity and sedentary behavior Tips for setting specific, measurable, achievable, relevant, time-bound (SMART) goals
5	Understanding food labels Limiting sugar-sweetened beverage consumption Improving sleep hygiene Avoiding too much sleep on weekends (“social jet lag”)
6	Associations and interrelations between health habits Relationships between the Big 6 and mental health Physical, social, and emotional benefits of health and well-being The Big 6 and long-term health

#### Character Development and Script Writing

The first step in designing the cartoon modules was to develop character profiles. The characters were purposely designed to be of similar age to the target audience (ie, grade 7 students, aged approximately 12 years) and to have different strengths and weaknesses and various health behavior clusters. Basic character profiles were developed with and reviewed by young people (n=7; aged 12-15 years; 2 males and 5 females) recruited via personal and professional networks. The youth reviewers were asked to make suggestions about character names, appearance, personalities, and health habits and to provide ideas for age-appropriate scenarios to be embedded into the storylines. Next, based on these characters and the findings from the web-based youth survey (stage 2), initial cartoon scripts for the 6 modules were written by members of the research team. The scripts aimed to provide students with information and skills about the Big 6 through the context of a teenage drama. The script writing was an iterative process, undergoing review by the expert researchers (n=22) and young people (n=9) before being sent for animation. Youth reviewers were asked to comment on the language used and relevance of the storylines, whereas expert reviewers commented on the accuracy of the content and prevention messages. Scripts were modified accordingly and then sent to animators to develop into the cartoon animations.

#### Module Summaries and Activities

Consistent with the *Climate Schools* programs [26-28], teacher and student summaries and optional class activities were developed, in addition to the cartoon storylines, for each of the 6 modules. Module summaries were developed to reinforce the key content taught within the cartoons. The summaries were written by members of the research team, drawing on the latest available evidence, including national prevalence data, for example [46,47], national guidelines, for example [48], and recent scientific literature relating to the Big 6 risk factors, for example, benefits of engaging in healthy behaviors and consequences of engaging in risky behaviors. In addition, a set of 4 to 5 activities was developed for each module, including points for class discussions, worksheets, quizzes, and homework tasks. Activities were designed to develop key self-management and interpersonal skills, such as communication, decision making, and problem-solving, and to meet key outcomes from the stage 4 New South Wales Personal Development, Health and Physical Education syllabus, the Western Australian Year 7 Health and Physical Education syllabus, and the years 7 and 8 Australian Health and Physical Education curriculum (for Queensland). Each module includes one web-based activity, such as interactive worksheets, games, or quizzes, to provide an engaging and immersive learning experience. Summaries and activities for each module were reviewed by members of the wider Health4Life team, including those with expertise relevant to the Big 6 behaviors.

### Stage 4: User Testing

User testing was conducted in 2019 with year 7 students and teachers to gain feedback on the acceptability and feasibility of the Health4Life school-based program before implementation. Ethical approval was obtained from the University of Sydney HREC (2018/882).

#### Student Testing: Participants and Procedure

A selection of secondary schools in Sydney that had previously expressed interest in participating in research were invited to participate in user testing of the Health4Life program, of which 2 agreed to participate. A total of 2 focus groups (45-60 min) were conducted with grade 7 students in 2 independent secondary schools in Sydney: one all male (n=21; mean age 12.05 years, SD 0.38) and one all female (n=20; mean age 12.10 years, SD 0.31). Consent forms were distributed to parent or guardians, with passive parental consent and active student consent required. Facilitated by 2 researchers, the focus group consisted of students watching a video presentation of modules 1 and 2 cartoons as a group. Following each cartoon, the facilitators led a class discussion, prompting students with questions regarding the likability and relatability of the characters, storylines, and language used in the cartoons. The focus groups were audio recorded and later transcribed. At the end of the discussion, students were also asked to complete a brief, 10-min questionnaire to provide their written feedback about the acceptability and relevance of the Health4Life modules. Students were remunerated a Aus $30 (US $20) gift voucher for their time spent in the focus group. Students were also invited to provide feedback on the remaining 4 modules via a web-based survey for an additional reimbursement of one Aus $15 gift (US $10) voucher per module.

#### Teacher Testing: Participants and Procedure

All grade 7 health education teachers at the same 2 participating schools were invited to participate in the user testing process. Additional teachers were recruited via social media and personal and professional networks. A total of 8 teachers from 5 schools agreed to participate. After providing informed consent, teachers were invited to view 2 Health4Life modules (including cartoons, module summaries, and activities) and asked to complete a web-based survey to comment on the acceptability and suitability of the Health4Life school-based program. The teachers were reimbursed Aus $50 (US $35) for each module they reviewed. In addition, health education curriculum experts (n=6) from New South Wales, Western Australia, and Queensland were engaged to provide expert advice on the alignment of the Health4Life content with the national- and state-based health education curricula.

#### Analysis

Student and teacher questionnaire data were analyzed using IBM SPSS Statistics 24. Descriptive statistics were conducted to calculate the percentage agreement for each survey item. The focus group data were transcribed by one researcher (EH). Individual comments and specific recommendations were extracted to inform refinements and modifications to the Health4Life program.

## Results

### User Testing Results

A total of 41 students (mean age 12 years, SD 0.35; 49% [20/41] female) provided feedback about the Health4Life school-based program. As the review of modules 3 to 6 occurred outside of the classroom and was optional, the response rate ranged from 39% (16/41) to 100% (41/41) across modules. Overall, students rated the cartoon modules favorably, providing positive feedback about the likability and believability of the storylines and characters (Table 5). An example of open-ended feedback provided by students is summarized in Multimedia Appendix 1.

**Table 5:** Summary of student questionnaire data.

Question	Module 1 (n=41), n (%)	Module 2 (n=41), n (%)	Module 3 (n=31), n (%)	Module 4 (n=26), n (%)	Module 5 (n=18), n (%)	Module 6 (n=16), n (%)
Overall rating (% good/very good)	36 (88)	37 (90)	30 (97)	24 (92)	16 (89)	14 (88)
How much did you like the storylines? (% liked a little/lot)	35 (85)	38 (93)	30 (97)	24 (92)	18 (100)	13 (81)
How much did you like the characters? (% liked a little/lot)	29 (71)	34 (83)	28 (90)	24 (92)	17 (94)	12 (75)
How believable and realistic were the storylines? (% completely/somewhat)	31 (78)	32 (82)	26 (87)	23 (88)	16 (89)	13 (81)
Do you think that other year 7 students will understand the information in the lessons? (% strongly agree/agree)	30 (77)	—^a^	22 (73)	22 (85)	14 (78)	14 (88)
Do you think other year 7 students will like the characters? (% strongly agree/agree)	28 (72)	—	20 (67)	20 (77)	14 (78)	11 (69)
Do you think that other students will find the cartoons an engaging way to learn? (% strongly agree/agree)	32 (82)	—	21 (70)	20 (77)	11 (61)	10 (63)

^a^Students were asked to respond to questions 5-7 about both modules 1 and 2, so separate data for each module were not available.

A total of 8 teachers (75% male) participated in user testing: 7 taught at coeducational schools and 1 taught at a single-sex school. On average, participants had been teaching for 11.5 (SD 10.09) years. Table 6 summarizes the feedback provided by teachers via the web-based questionnaire. Overall, the teachers rated the Health4Life program positively, with the percentage of teachers rating the modules as good or very good ranging from 88% to 100%. The vast majority of teachers also indicated that the modules were a good fit with the health education syllabus and that the storylines were believable, age appropriate, and understandable to students. However, some teachers had concerns about the modules adequately covering content, having sufficient lesson time, and feasibility of implementation. Multimedia Appendix 2 provides key themes and examples extracted from the open-ended teacher feedback.

**Table 6:** Summary of teacher questionnaire feedback.

Feedback item	Module 1 (n=8), n (%)	Module 2 (n=8), n (%)	Module 3 (n=7), n (%)	Module 4 (n=7), n (%)	Module 5 (n=7), n (%)	Module 6 (n=7), n (%)
Overall rating (% good/very good)	7 (88)	7 (88)	7 (100)	7 (100)	7 (100)	7 (100)
Fit with syllabus (% very well/extremely well)	6 (75)	6 (75)	6 (86)	6 (86)	7 (100)	7 (100)
Believable and valid storylines (% yes)	7 (88)	8 (100)	7 (100)	7 (100)	6 (86)	7 (100)
Age-appropriate content (% yes)	8 (100)	7 (88)	7 (100)	7 (100)	7 (100)	7 (100)
Concepts understood/remembered by students (% yes)	8 (100)	8 (100)	6 (86)	6 (86)	7 (100)	7 (100)
Lesson length adequately covers content (% yes)	4 (50)	5 (63)	3 (43)	4 (57)	4 (57)	3 (43)
Language acceptable to youth (% yes)	8 (100)	7 (88)	7 (100)	7 (100)	7 (100)	7 (100)
Concerns about language used (% yes)	1 (13)	1 (13)	1 (14)	1 (14)	0 (0)	0 (0)
Effectiveness of program improving students’ health behaviors (% somewhat effective/very effective)	8 (100)	8 (100)	7 (100)	7 (100)	7 (100)	7 (100)
Likely to have implementation problems (% yes)	5 (63)	5 (63)	4 (57)	4 (57)	1 (14)	1 (14)
Sufficient time to deliver modules (% yes)	6 (75)	6 (75)	4 (57)	4 (57)	4 (57)	4 (57)
Sufficient computers for students (% yes)	7 (88)	7 (88)	6 (86)	6 (86)	5 (71)	5 (71)

#### Key Changes Made to the Health4Life Program After User Testing

On the basis of feedback obtained from students and teachers, several modifications were made to the Health4Life program, including changes to the language, a reduction in cartoon text and slides, the addition of a backstory module and links to syllabus overviews, and the provision of alternate delivery methods (Table 7).

**Table 7:** Examples modifications made to the Health4Life program.

Key issues identified	Modifications
Lesson length Text-heavy cartoons Too many cartoon slides Concern over fitting the cartoons and activities into one lesson	Scripts were revised to make wording more succinct, and cartoon slides were removed where possible. It was made clear to teachers that the class activities are optional and that a range of activities are provided so that the most suitable and feasible options can be selected.
Cartoon content Age appropriateness of language Need for a backstory Relatability of alcohol storyline for year 7 students	Language was refined based on student suggestions (eg, changing “okay” to “kk” in text messages). An introductory cartoon module was added to provide a backstory for each of the main characters. The script was adapted to make it clear that, aside from Xavier (year 7), the only other characters drinking alcohol are older (year 10).
Linking content to the health and physical education curricula	Expert curriculum consultants were engaged to develop or review unit overviews that map the cartoon content and activities to the relevant syllabus outcomes and descriptors for each state. An outline of how the components of the Health4Life program align with the different stages of learning (knowledge, understanding, skills, and application) and the 5 propositions (taking a strengths-based approach, focusing on educative purposes, valuing movement, developing health literacy, and including a critical inquiry approach) that shape the health and physical education curricula was provided.
Implementation feasibility for schools with limited access to computer rooms or devices	Teachers were provided with alternative delivery options, such as using a smart board to go through the modules as a class or using hard copies of the cartoons and activities.

#### The Final Health4Life School-Based Program

**Figure 2:**
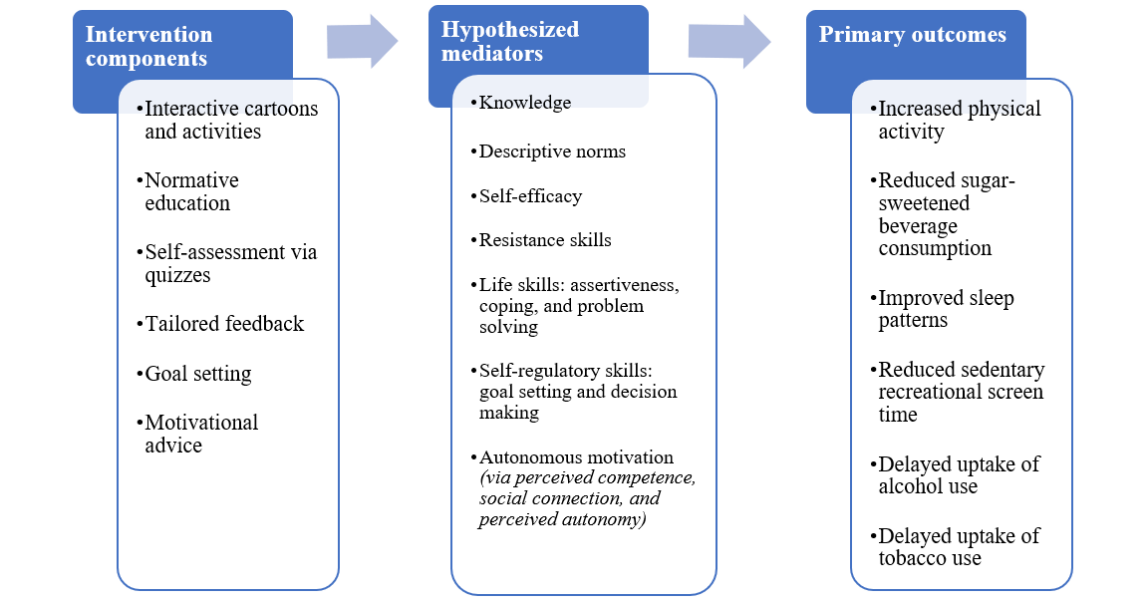
Health4Life Conceptual model.

The Health4Life school-based program is underpinned by a multiple health behavior change approach [10]. It is anticipated that by providing students with concurrent education about the Big 6, while also highlighting the associations and interrelations between health behaviors, the program will efficiently facilitate change across multiple behaviors. The program uses principles of social influence [29], social cognitive [49], social learning [50] and self-determination theories [51], and the two-process model of sleep [52,53] (Table 1 and Figure 2), with key behavior change components and mechanisms for change woven into the storyline and accompanying activities. The Health4Life school-based program was developed to meet outcomes from the stage 4 New South Wales Personal Development, Health and Physical Education syllabus, the Western Australian Year 7 Health and Physical Education syllabus, and the years 7 and 8 Australian Health and Physical Education curriculum (for Queensland). Teachers are provided with unit overviews that map the cartoon content and activities to the state-based syllabus outcomes and descriptors, along with an outline of how the components of the Health4Life program align with the different stages of learning (knowledge, understanding, skills, and application) and the 5 propositions (taking a strengths-based approach, focusing on educative purposes, valuing movement, developing health literacy, and including a critical inquiry approach) that shape the health and physical education curriculum. An overview of the program content is provided in Table 4.

**Figure 3:**
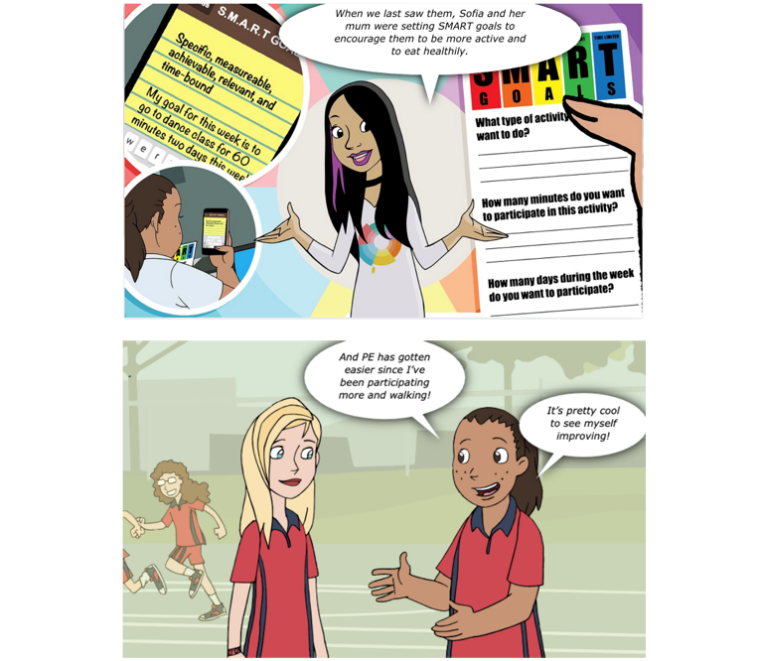
Example cartoons from the Health4Life modules.

The Health4Life school-based program consists of 6 modules, ideally delivered once per week, each comprising the following:

1. A 20-min web-based cartoon completed individually by students (Figure 3): The cartoons follow the story of a group of teenagers, of similar ages to grade 7 students, to impart key prevention messages while engaging students and maintaining interest. The cartoon modules aim to provide evidence-based information about the Big 6, improve resistance skills, modify existing norms, and increase autonomous motivation. Short web-based quizzes are embedded at the end of each cartoon module to test knowledge.
2. Module summaries: teachers and students are provided with PDF factsheets for each module to provide additional details and reinforce key messages.
3. Optional activities: teachers are provided with a selection of 4 activities (eg, worksheets, group discussions, and homework tasks) to implement with their students after the cartoon in remaining lesson time (approximately 20 min). Teachers can select which and how many activities they implement to best suit the needs of their class. A self-directed interactive web-based activity (eg, web-based worksheet and game) is also included after each cartoon module to accommodate students completing the cartoon component at different speeds.

**Figure 4:**
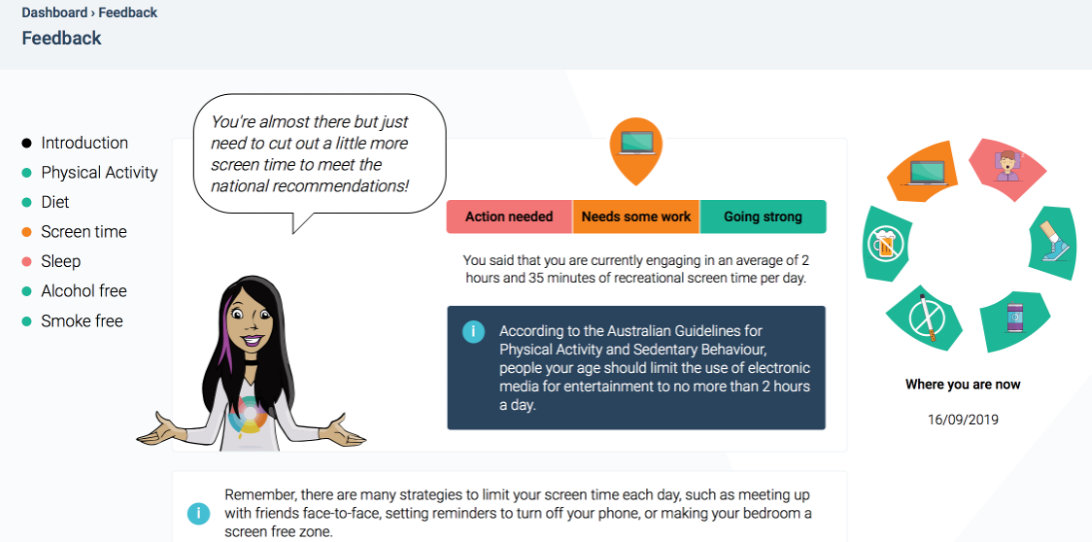
Example web-based tailored feedback.

On the basis of the findings of the systematic review conducted in stage 1, the final component of the Health4Life school-based program is web-based tailored feedback. Students are provided color-coded strengths-based feedback about their adherence to national health guidelines for each of the Big 6 immediately after completing a self-report web-based assessment, via the study website (Figure 4). The feedback aims to help students identify behaviors to increase, decrease, or maintain. Using a traffic light system, *green* notifications are provided to students who are currently meeting national guidelines (*Going Strong*), *orange* notifications are given to students who are not yet meeting guidelines but are close (*Needs some work*), and *red* notifications are provided to students who are not meeting guidelines (*Action needed*).

#### Program Implementation

The Health4Life school-based program is a universal intervention designed to be delivered to all grade 7 students, regardless of their level of risk for chronic disease. It was designed for delivery during health education classes approximately once per week over 6 weeks. All materials are available on the web and accessed via student and teacher portals. Teachers are provided with implementation guidelines and links to the syllabus; however, no teacher training is required.

## Discussion

### Principal Findings

This study aimed to describe the comprehensive, formative research and development process of the school-based component of the *Health4Life* initiative. The program was developed in collaboration with students, teachers, and health professionals and is grounded in theory and the best available scientific evidence and is aligned closely with the latest health education curricula in Australia.

### Strengths and Limitations

A key strength of the Health4Life school-based program is that it is the first eHealth intervention to concurrently address the Big 6 lifestyle risk factors for chronic disease—physical inactivity, poor diet, smoking, alcohol use, poor sleep, and sedentary recreational screen time—among school students. By adopting a multiple health behavior change approach, it has the potential to efficiently modify the Big 6 within one program and to equip young people with the skills and knowledge needed to maintain good physical and mental health throughout adolescence and into adulthood. Although not the focus of this paper, the Health4Life school-based program is supplemented by a universal smartphone app that prompts students to monitor their behaviors, track their progress, and set goals. In addition, a selective intervention based on cognitive behavioral and motivational enhancement principles provides youth who remain at risk of chronic disease when they are in grades 8 and 9, with additional web and app content to assist them in developing coping strategies and skills to facilitate healthy behavior change. Detailed information about these additional components has been published elsewhere [54]. Together, these 3 components span both universal and selective prevention, maximizing outcomes for students throughout their secondary school years. Finally, as the Health4Life program is delivered to students via web-based technology with interactive components and no teacher training is required for implementation, student engagement and program fidelity is likely to be increased [21] (assessment of these outcomes is reported on elsewhere [54]). Importantly, web-based interventions also typically lead to improved scalability and sustainability, which enhances the dissemination potential and reach of the Health4Life program. A notable limitation of the co-design process is that students and teachers were only recruited from independent schools in Sydney for user testing and, although the web-based survey sample in stage 2 spanned across 2 states, participants were predominantly female. Nonetheless, this study was successful in engaging end users at various stages of the development process, and the final program was rated favorably and deemed acceptable and relevant by young people.

### Future Directions

The next important step is to evaluate the effectiveness of the Health4Life intervention. A large cluster randomized controlled trial is currently underway in 71 Australian schools (>6600 students) to evaluate whether Health4Life is more effective than health education as usual in delaying the uptake of alcohol and tobacco use, reducing sedentary recreational screen time, reducing the decline in MVPA, reducing consumption of sugar-sweetened beverages, and improving sleep [54].

### Conclusions

Ultimately, this study has the potential to make a substantial public health impact by concurrently addressing 6 key risk factors, thereby reducing the incidence of chronic disease and minimizing the associated costs, disability, and early mortality.

## Appendix

Multimedia Appendix 1 Student open-ended feedback.

Multimedia Appendix 2 Teacher open-ended feedback.

## Abbreviations

eHealth: electronic health
HREC: human research ethics committee
MVPA: moderate-to-vigorous physical activity
SMD: standard mean difference
