# Supplementary material for: A Web-Based Intervention to Prevent Multiple Chronic Disease Risk Factors Among Adolescents: Co-Design and User Testing of the Health4Life School-Based Program
Source: JMIR Form Res. 2020 Jul 28;4(7):e19485. doi: 10.2196/19485 (PMC7420628; doi:10.2196/19485)
Supplement: Multimedia Appendix 1 [file formative_v4i7e19485_app1.docx]

Multimedia Appendix 1: Student open-ended feedback

| **Strengths** | **Areas for Improvement** |
| --- | --- |
| **Overall feedback** | |
| - “I like the way that the modules were presented as cartoons” - “I liked every part- very relatable” - “I liked how it showed the effects of drinking alcohol” - “I liked how it linked very well to our lives” | - “There were too many slides” - “Some of the speech bubbles had really long paragraphs that made it hard to read” - “I liked everything except the kids drinking, as kids our age don’t drink” |
| **Character Feedback** | |
| - “There are no changes I would make as they were fun and cool to watch” - “No changes, all of the characters were good” | - “They should have a bit of a back story” - “The language used by the characters isn't the language kids of that age use” - “I would try and make them more relatable to our age group” |
| **Storyline Feedback/ Believability of Storylines** | |
| - I don’t think there are any changes that I would make to the story” - “I would make no changes to the storylines” - “I liked how Izzy had her phone taken away, that was realistic” - “No changes needed to make the storylines more believable or realistic” | - “I would make the modules a little more realistic” - “Maybe give more details to the events like the alcohol drinking” - “Some of the character development happened too easily, like Xavier deciding that drinking wasn't required to have fun” |
| **Other Feedback** | |
| - “I think that this is a very fun and simple way to learn” - “I liked that it was very informative and gave a lot of information” - “I really liked how many girls my age would be able to relate to this” - “I like that the students were given an informative lesson about ‘The Big 6’ in the last module” | - “Maybe change the excuse from "my dad will kill me" to "my dad will be as angry as a lion," etc. I feel like 'kill' is a bit too strong of a word” - “Maybe make it an audio instead of a slide show, but overall it was good” - “Less focus on getting drunk and more focus on phone addiction” |
