# Supplementary material for: A Web-Based Intervention to Prevent Multiple Chronic Disease Risk Factors Among Adolescents: Co-Design and User Testing of the Health4Life School-Based Program
Source: JMIR Form Res. 2020 Jul 28;4(7):e19485. doi: 10.2196/19485 (PMC7420628; doi:10.2196/19485)
Supplement: Multimedia Appendix 2 [file formative_v4i7e19485_app2.docx]

**Multimedia Appendix 2:** Teacher open-ended feedback

| **Strengths** | **Areas for Improvement** |
| --- | --- |
| **First Impressions** | |
| - “I think the cartoons are very engaging. Multiple issues are being brought up in one conversation. Traditionally, these were taught individually” - “I think it looks really good. The lessons look age appropriate and engaging. It has strong links to the new curriculum” | - “The cartoons were fairly long, not sure some classes would get through it in 20 minutes” |
| **What did you like?** | |
| - “I thought it was useful to present the information/context for the lessons in a cartoon. This way students could easily relate to some of the situations they may have encountered. This allows students to project themselves in challenges they may face as teenagers by it ‘coming to life’ in a cartoon” - “A range of different activities used throughout the lesson e.g. literacy task + discussion which is catering for a range of students” | - “No syllabus dot points linked in any of the teacher material” - “Lots of characters. It may be hard for students to remember” - “The lessons are very content heavy and it may not be realistic to get through that amount of content in one lesson” |
| **Links to Syllabus** | |
| - “I think it suits the NSW syllabus very well” | - “It would be good to include the outcomes in the teacher resources, so it's clear what outcomes are being met” - “Incorporate a strengths-based approach” - “Lack of content being linked to specific outcomes in syllabus” |
| **Believability and Relatability** | |
| - “The cartoons presented realistic scenarios for students in Year 7. They weren't too far-fetched and they focused on a range of health concepts and behaviours that they are most likely to encounter as they start high school” - “The characters have a variety of personalities and traits that most students will recognise having some similarities to themselves. Regardless of whether they find themselves in similar scenarios, they can relate to the characters and their circumstances” | - “I think the language is fine e.g. text messages etc., but there is the assumption that this age group are going to be involved in the social aspect of drinking etc. and I don't think that is the case in a lot of schools” - “The behaviours explored are common for young adolescents. Peer pressure around alcohol and smoking are the behaviours which some students might find it harder to relate to” |
| **Appropriateness of Educational Content** | |
| - “The content is reasonable and appropriate for year 7. The big 6 are extremely relevant at this age” | - “Technology/ social media dependence is very appropriate and relevant, but the alcohol scenario would be less so for many year 7 students” |
| **Student Ability to Understand and Remember Concepts Taught** | |
| - “The concepts are pitched at a level that year 7 students can understand. I think gifted students will find this lesson very simple” - “Concepts are simple and the cartoon uses easy to remember examples that are relatable” | - “I think more time is needed on each of the focus areas. I think lessons move through the content too quickly for students to retain their understanding” - “There are quite a few characters - I think less characters would help students be able to recall scenarios from the cartoon” |
| **Lesson Length** | |
| - “Year 7 students attention span is short, therefore keeping the lessons to 20 - 40-minute blocks allows them focus/listen more attentively” - “Time is appropriate for the level of work required/slides they must view and activities.  I would give for homework if not complete. This meets the average level of ability” | - “The content moves too quickly for some lower ability students, particularly if the student has little or no prior understanding about the content” - “I think the activities may be too in-depth to cover a 20-minute period” - “I don’t think students will be able to read the scenarios in 20 minutes” |
| **Acceptability of Language** | |
| - “The language used is very appropriate for year 7 students” | - “Could incorporate some shorten slang words i.e. Lol, Fomo (fear of missing out) in certain parts of the cartoon to increase students relating to the cartoons” - “My only concern would be for NESB (non-English speaking background) students who may struggle with some of the language” |
| **Perceived Effectiveness of Program** | |
| - “I believe the program will be effective in engaging the majority of year 7 students, as most students will be able to relate to the scenarios and characters established in the cartoons” | - “I think it is very difficult to change lifestyle behaviour without the support of the parents. You would need to supplement this with an information pack that the parents could access” |
| **Time Proposed to Deliver Modules** | |
| - “I think 6 lessons is very achievable for this unit” - “My school has 55-minute lessons and students are selective. They would potentially move through the activities within that time” - “If I could justify that outcomes are being met, six lessons is not a large amount. We use other online courses that are similar in length” | - “Difficult to know” - “Only if units are adjusted” - “I do think that it would take longer than the time you have suggested with most Year 7 classes” - “I think it would take longer than 1 lesson to get through the lesson and activities” |
| **Implementation Feasibility** | |
| - “Do not need internet to use pdf cartoons” - “Potential issues with laptop accessibility, however the pdfs can be printed out” | - “Students may not have a device” - “As content is covered in our programs which are carefully planned across stage 4, it would be hard to just slip this in without doubling up. If a unit was built around this resource, that would be better” - “Finding space/ time in current units may be difficult” |
